# Targeting interferon responses in juvenile dermatomyositis: Siglec-1 as an in vitro biomarker for JAK inhibitor efficacy

**DOI:** 10.1093/rheumatology/keaf227

**Published:** 2025-05-15

**Authors:** Saskia R Veldkamp, Maud Reugebrink, Sanne W Evers, Thomas R J Moreau, Vincent Bondet, Wineke Armbrust, J Merlijn van den Berg, Petra C E Hissink Muller, Sylvia Kamphuis, Ellen Schatorjé, Eveline M Delemarre, Anneke J van der Kooi, Brigitte Bader-Meunier, Darragh Duffy, Mathieu P Rodero, Joost Raaphorst, Annet van Royen-Kerkhof, Marc H A Jansen, Femke van Wijk

**Affiliations:** Center for Translational Immunology, University Medical Center Utrecht, Utrecht, The Netherlands; Center for Translational Immunology, University Medical Center Utrecht, Utrecht, The Netherlands; Department of Neurology, Amsterdam University Medical Centre, Location AMC, Amsterdam, The Netherlands; Université Paris Cité, CNRS, Laboratoire de Chimie et de Biochimie Pharmacologiques et Toxicologiques UMR8601, Paris, France; Translational Immunology Unit, Institut Pasteur, Université Paris Cité, Paris, France; Translational Immunology Unit, Institut Pasteur, Université Paris Cité, Paris, France; Department of Paediatric Rheumatology and Immunology, Beatrix Children’s Hospital, University Medical Center Groningen, Groningen, The Netherlands; Department of Paediatric Immunology, Rheumatology and Infectious Diseases, Emma Children’s Hospital, Amsterdam University Medical Centre, Location AMC, Amsterdam, The Netherlands; Department of Paediatric Rheumatology, Willem Alexander Children’s Hospital, Leiden University Medical Centre, Leiden, The Netherlands; Department of Paediatric Rheumatology, Sophia Children’s Hospital, Erasmus University Medical Centre, Rotterdam, The Netherlands; Department of Paediatric Rheumatology, Amalia Children’s Hospital, Radboud University Medical Centre, Nijmegen, The Netherlands; Center for Translational Immunology, University Medical Center Utrecht, Utrecht, The Netherlands; Department of Neurology, Amsterdam University Medical Centre, Location AMC, Amsterdam, The Netherlands; Paediatric Haematology-Immunology and Rheumatology Department, Necker-Enfants Malades Hospital, AP-HP, Paris, France; Translational Immunology Unit, Institut Pasteur, Université Paris Cité, Paris, France; Université Paris Cité, CNRS, Laboratoire de Chimie et de Biochimie Pharmacologiques et Toxicologiques UMR8601, Paris, France; Department of Neurology, Amsterdam University Medical Centre, Location AMC, Amsterdam, The Netherlands; Department of Paediatric Rheumatology and Immunology, Wilhelmina Children’s Hospital, University Medical Center Utrecht, Utrecht, The Netherlands; Department of Paediatric Rheumatology and Immunology, Wilhelmina Children’s Hospital, University Medical Center Utrecht, Utrecht, The Netherlands; Center for Translational Immunology, University Medical Center Utrecht, Utrecht, The Netherlands

**Keywords:** interferon, JAK inhibition, Siglec-1, juvenile dermatomyositis, assay, precision treatment

## Abstract

**Objectives:**

For IFN-driven diseases, such as juvenile dermatomyositis (JDM), there is a critical need for targeted therapies. We aimed to develop an *in vitro* model, using Siglec-1 as read-out, to evaluate inhibition of IFN-mediated responses with different JAK inhibitors (JAKi).

**Methods:**

Healthy donor (HD) PBMCs were cultured with type I and II IFNs, TLR agonists and plasma or serum from patients (JDM, DM, JSLE, COVID-19) and HDs. Siglec-1 expression on CD14^+^ monocytes was analyzed using flow cytometry. Inhibitory assays involved pre-incubation with JAKi (filgotinib, tofacitinib, baricitinib, ruxolitinib, deucravacitinib) and interferon-α/β receptor (IFNAR)-blocking antibody. Correlations between plasma-induced Siglec-1 levels and clinical disease activity were analyzed in JDM patients, as well as correlations with IFN-α and -β plasma levels.

**Results:**

Siglec-1 was induced after 18 h of stimulation with type I IFNs and TLR-3/7/9 agonists, with minimal induction by IFN-γ. IFNAR blockade prevented type I IFN- and TLR-mediated induction. JAKi inhibited Siglec-1 induction by IFN-α and -β in a dose-dependent manner. Co-culture with plasma or serum from patients with IFN-driven diseases induced Siglec-1 expression on healthy monocytes, which could be inhibited by JAKi and IFNAR blockade. Siglec-1 levels induced by JDM plasma correlated strongly with clinical disease activity and IFN-β plasma levels.

**Conclusion:**

Siglec-1 is an easy and reliable *in vitro* marker for type I IFN responses. Its induction can be inhibited by JAKi. The type I IFN signature in JDM is likely predominantly driven by IFN-β. This assay holds promise for precision treatment strategies in JDM and other IFN-driven diseases.

Rheumatology key messages

- Siglec-1 expression, an accurate IFN-I read-out, was inhibited by JAKi in a dose-dependent manner.
- Co-cultures with JDM plasma induced Siglec-1, correlating strongly with disease activity and IFN-β plasma levels.
- This assay shows potential for guiding JDM patient stratification and optimizing JAKi treatment selection.

## Introduction

Juvenile dermatomyositis (JDM) is a rare paediatric immune-mediated inflammatory disease typically characterized by symmetrical proximal muscle weakness and a pathognomonic skin rash. Other vital organs such as the lungs, heart and intestines can also be affected. Current standard treatment includes a combination of high-dose steroids and methotrexate, with escalation or modification of therapy for resistant disease [1]. However, treatment response is variable and unpredictable, with up to two-thirds of patients having a chronic or polycyclic disease course [2, 3]. These patients are at risk for long-term damage, including calcinosis, joint contractures and growth delay, which is related to both ongoing disease activity and toxicity from corticosteroids [2, 4]. Therefore, there is a significant unmet need for developing a more targeted treatment approach, for which a better understanding of disease mechanisms is essential [3].

**Figure 1.**
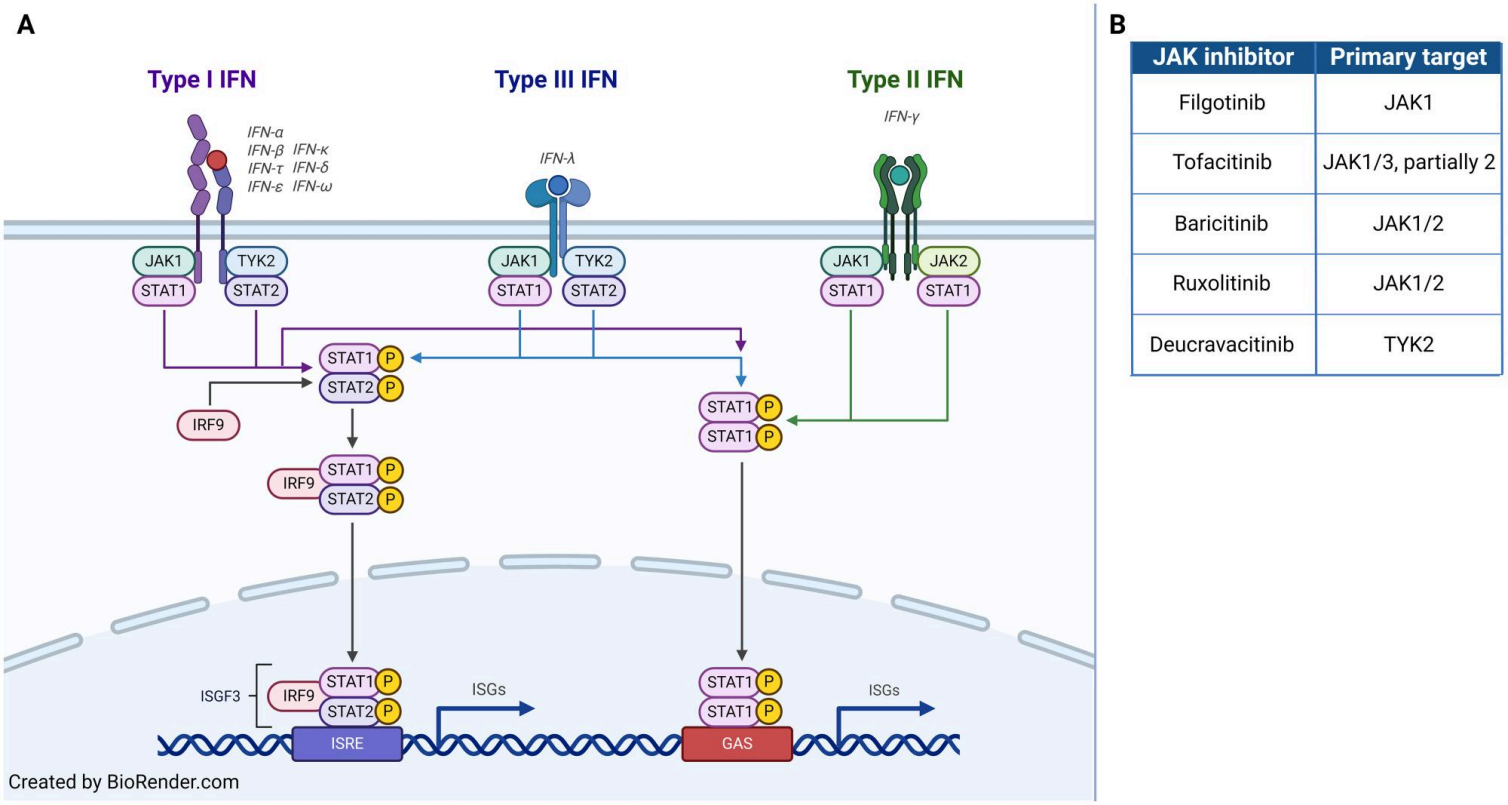
Interferon pathway and JAK inhibitors. (A) Illustration of the IFN pathway (BioRender 2019). Different types of IFN bind to their specific receptors, activating the Janus Kinase/signal transducer and activator of transcription (JAK/STAT) signalling pathway and resulting in transcription of IFN-stimulated genes (ISGs). Notably, the JAKs and STATs are also utilized by other cytokine signalling pathways. (B) The primary targets of the JAK inhibitors used in the current study. IRF = interferon regulatory factor, ISRE = interferon-sensitive response element; GAS = interferon-gamma-activated site

A dysregulated IFN pathway is considered central in disease pathogenesis. An upregulated IFN signature has been detected at both gene expression and protein levels in peripheral blood and tissue, and correlates with clinical disease activity [5–7]. Although most research has focused on the role of type I IFNs, and primarily on IFN-α and -β, there is evidence that type II IFN (IFN-γ) may also be involved [3, 8–10] (Fig. 1A). Recent research has demonstrated that Siglec-1 (sialic acid-binding Ig-like lectin 1), an IFN-induced marker, correlates with clinical disease activity in JDM patients [11]. Additionally, it has the potential to identify patients at an increased risk for needing treatment intensification within the first three months after diagnosis [11]. Siglec-1, also known as Sialoadhesin or CD169, is an adhesion molecule present on the surface of macrophages and activated monocytes that can bind to various immune cells and activated endothelial cells [12, 13]. Besides JDM, increased Siglec-1 expression has also been observed in other IFN-driven diseases such as SLE, primary SS, SSc and adult DM [14–18].

Because of the well-established IFN signature, there is growing interest in novel therapeutics targeting the IFN pathway for treatment of JDM. Janus kinase inhibitors (JAKi) are increasingly being used to treat patients with refractory JDM, demonstrating favourable outcomes in both muscle and skin disease management [19]. While these advancements are promising, critical questions remain regarding the optimal choice of JAKi, as well as the timing and patient selection for treatment. JAKi differ in their selectivity for JAK isoforms and their binding mechanisms. For instance, filgotinib has demonstrated preferential inhibition of JAK1, whereas tofacitinib is considered a JAK1/3 and partial JAK2 inhibitor (Fig. 1B). While the classical JAK1-3 inhibitors are ATP-competitive inhibitors, deucravacitinib (a selective TYK2-inhibitor) represents the first approved allosteric inhibitor, binding to the pseudokinase domain. These distinctions may be relevant for optimal treatment efficacy and safety.

In the current study, our aim was to develop an *in vitro* model to study the inhibition of IFN-mediated responses, using different JAKi with Siglec-1 as the primary read-out. We stimulated healthy donor peripheral blood mononuclear cells (PBMCs) with various IFNs, cytokines and Toll-like receptor (TLR) agonists and with plasma or serum collected from patients with different IFN-driven diseases, including JDM, adult DM, JSLE and COVID-19. We studied the inhibitory potency of different JAKi, as well as correlations between Siglec-1 induction and clinical disease activity.

## Methods

### Patients and healthy donors

Peripheral blood samples were obtained from 24 healthy donors (HDs) and from 18 patients with various IFN-driven diseases. Of the 19 HDs from whom we used PBMCs, 12 were female (63%) and the median age was 36 (IQR 30–55). Of the five HDs from whom we used plasma, age and sex are noted in Table 1. Out of eight patients with JDM, seven fulfilled the Bohan and Peter diagnostic criteria and ACR/EULAR classification criteria for JDM and one was diagnosed with “JDM sine dermatitis” [20–22]. All six adult DM patients had biopsy-proven DM based on the 2004 European Neuromuscular Centre (ENMC) criteria [23]. Both JSLE patients met the SLICC classification criteria for SLE [24]. The two adults with COVID-19 were sampled on the day of hospitalization and were classified with “severe COVID-19” based on the WHO COVID-19 severity classification [25].

**Table 1.** Characteristics of patients and HDs from whom plasma or serum was used

Patient/HD	Age at sampling (years)	Sex (male/female)	Disease activity status
*JDM diagnosis*			
JDM1	12	F	PGA-VAS 8
JDM2	7	M	PGA-VAS 7
JDM3	5	M	PGA-VAS 7
JDM4	5	M	PGA-VAS 5
JDM5	4	F	PGA-VAS 3
JDM6	9	F	PGA-VAS 5
JDM7	5	F	PGA-VAS 8
JDM8	8	M	PGA-VAS 6
*JDM remission*			
JDM1	14	F	PGA-VAS 0 (under MTX)
JDM2	8	M	PGA-VAS 0 (under MTX)
*DM diagnosis*			
DM1	37	M	
DM2	30	F	
DM3	59	M	
DM4	34	F	
DM5	67	F	
DM6	25	M	
*JSLE diagnosis*			
SLE1	15	M	SLEDAI 10
SLE2	15	F	SLEDAI 10
*COVID-19 hospitalization*			
COVID1	37	F	Severe COVID-19^a^, 5 days hospital admission, no ICU
COVID2	55	F	Severe COVID-19^a^, 5 days hospital admission, no ICU
*HD*			
HD1	8	M	
HD2	55	F	
HD3	40	F	
HD4	44	F	
HD5	44	F	

a Based on the WHO COVID-19 severity classification. HD = healthy donor, F = female, M = male, PGA-VAS = Physician’s Global Assessment of overall disease activity measured on a Visual Analogue Scale (0–10), MTX = methotrexate, SLEDAI = Systemic Lupus Erythematosus Disease Activity Index (0–105), ICU = intensive care unit.

### Ethics

This study was approved by the medical ethics committee of the University Medical Center Utrecht (METC no. 15–191 and 07–025) and by the medical ethics committee of the Amsterdam University Medical Center (METC no. 2016_326). All patients and/or their parents gave their written informed consent in accordance with the declaration of Helsinki. The COVID-19 patient samples were obtained under study protocol TCbio no.20–175, for which a waiver for formal ethical approval was provided by the medical ethics committee of University Medical Center Utrecht.

### Sample collection

Peripheral blood was obtained in sodium-heparin tubes for PBMC and plasma collection and in serum tubes for serum collection. Plasma and serum were spun down and aliquoted within 4 hours (h) after collection, and subsequently stored at −80°C until further use. PBMCs were isolated using Ficoll-Paque™ PLUS (GE 110 Healthcare) density centrifugation and stored in liquid nitrogen for long-term storage or at −80°C for short-term storage, until further use.

### In vitro stimulation and inhibition

Thawed PBMCs from HDs were plated in a 96-wells plate (2.0–2.5*10^5^ cells/well) in RPMI 1640 medium with L-glutamine, penicillin/streptomycin and 10% fetal calf serum (FCS), and treated for 6, 18, 48 and 72 h with one of the following ligands: human IFN-α 2a (type I IFN) (1000 U/ml; PBL), human IFN-β 1a (type I IFN) (1000 U/ml; PBL), IFN-γ (type II IFN) (10 ng/ml; R&D), TLR-3/MDA-5/RIG-I agonist poly I:C (50 μg/ml; high molecular weight, InvivoGen), TLR-7 agonist imiquimod (R837) (5 μg/ml; InvivoGen), TLR-9 agonist class A CpG oligonucleotides (CpG-A ODN) (1 μg/ml; InvivoGen), TLR-4 agonist LPS (10 ng/ml; InvivoGen), IL-1β (10 ng/ml; R&D) and TNF-α (10 ng/ml; Miltenyi). Siglec-1 expression was measured on CD14^+^ monocytes with flow cytometry. In inhibitory assays, PBMCs were pre-incubated for 1 h with one of the following inhibitors: anti-IFNα/βR2 blocking antibody (2 μg/ml; PBL, Clone MMHAR-2), JAK1-inhibitor filgotinib (0.1–10 μM; Selleckchem), JAK1/2/3-inhibitor tofacitinib (0.1–10 μM; Selleckchem), JAK1/2-inhibitor baricitinib (0.1–10 μM; Selleckchem), JAK1/2-inhibitor ruxolitinib (0.1–10 μM; Selleckchem), TYK2-inhibitor deucravacitinib (0.1–10 μM; Cayman chemical). After pre-incubation, PBMCs were stimulated with IFN-α (1000 U/ml) or IFN-β (1000 U/ml) for 18 h, followed by Siglec-1 expression analysis on CD14^+^ monocytes with flow cytometry. To assess the effects of plasma or serum, HD PBMCs were treated for 18 h with plasma or serum from patients or from (other) HDs (17% v/v), with or without 1 h pre-incubation with anti-IFNα/βR2 blocking antibody (2 μg/ml), deucravacitinib (0.1 μM) or baricitinib (1 μM). Plasma and serum were not subjected to heat inactivation. Control conditions were included in all experiments (unstimulated, dimethylsulphoxide (DMSO)-only, stimulus-only, inhibitor-only).

### Flow cytometry

PBMCs were thawed for *ex vivo* analysis or washed with PBS after *in vitro* culture, and subsequently stained with fixable viability dye eFluor780 (eBioscience™) for 25 min at 4°C. Cells were washed with PBS, followed by surface staining with the antibodies V500-conjugated anti-human CD14 (clone M5E2, BD) and PE-conjugated anti-human Siglec-1 (CD169) (clone 7–239, Thermo Fisher Scientific) for 20 min at 4°C in PBS (Sigma) containing 2% FCS, 0.1% NaN_3_ (Severn Biotech Ltd) and 2% normal mouse serum (Fitzgerald). Cells were washed twice with PBS, fixed with 1:3 Fixation/Permeabilization concentrate and Fixation/Permeabilization diluent (Invitrogen) for 30 min at 4°C, washed twice with Permeabilization Buffer (Invitrogen) and stored in FACS buffer until analysis. Siglec-1 expression was measured on viable CD14^+^ cells (mostly representing monocytes) with the BD LSRFortessa™ flow cytometer (BD Bioscience). Stringent washing steps and gating strategies were applied to minimize potential interference from soluble factors such as soluble Siglec-1 in supernatant, plasma or serum. The gating strategy is depicted in Supplementary Fig. S1, available at *Rheumatology* online. The Siglec-1 gate was set based on fluorescence-minus-one (FMO) controls.

### Simoa digital ELISA

Plasma samples from JDM patients were analyzed for IFN protein levels by a multiplexed Quanterix homebrew Simoa assay (digital ELISA). These assays quantified all 12 IFN-α subtype proteins (pan-IFN-α) and IFN-β simultaneously according to the manufacturer’s instructions and methods previously described [7, 26]. The limit of detection (LOD) for pan-IFN-α was calculated at 1.08 fg/ml while the LOD for IFN-β was at 0.47 pg/ml. For reference, 1 fg/ml of pan-IFN-α corresponds to 2.18 × 10^−4^ U/ml and 1 pg/ml of IFN-β corresponds to 0.412 U/ml.

### Data analysis

The data were analyzed by FlowJo V10 software (FlowJo LLC; BD Bioscience) and SPSS (IBM SPSS statistics version 27). Surface expression of Siglec-1 was presented as the percentage of Siglec-1^+^ cells within the viable CD14^+^ monocyte population (“Siglec-1 percentage”) or as the median fluorescent intensity (MFI) of Siglec-1 on viable CD14^+^ monocytes (“Siglec-1 MFI”). Differences in Siglec-1 expression between conditions were examined with the Wilcoxon matched-pairs signed ranked test with a minimum of *N* = 5 per condition. For correlations, the Spearman correlation coefficient (rs) was calculated. A two-sided alpha level of 0.05 was considered statistically significant. As a measure for clinical disease activity in JDM, the Physician’s Global Assessment of overall disease activity measured on a Visual Analogue Scale (PGA-VAS) score (0–10, with higher scores indicating more overall disease activity) was obtained. For JSLE patients, the Systemic Lupus Erythematosus Disease Activity Index (SLEDAI) score (0–105, with higher scores indicating more disease activity) was obtained.

## Results

### Siglec-1 is induced after 18 h of type I IFN stimulation

**Figure 2.**
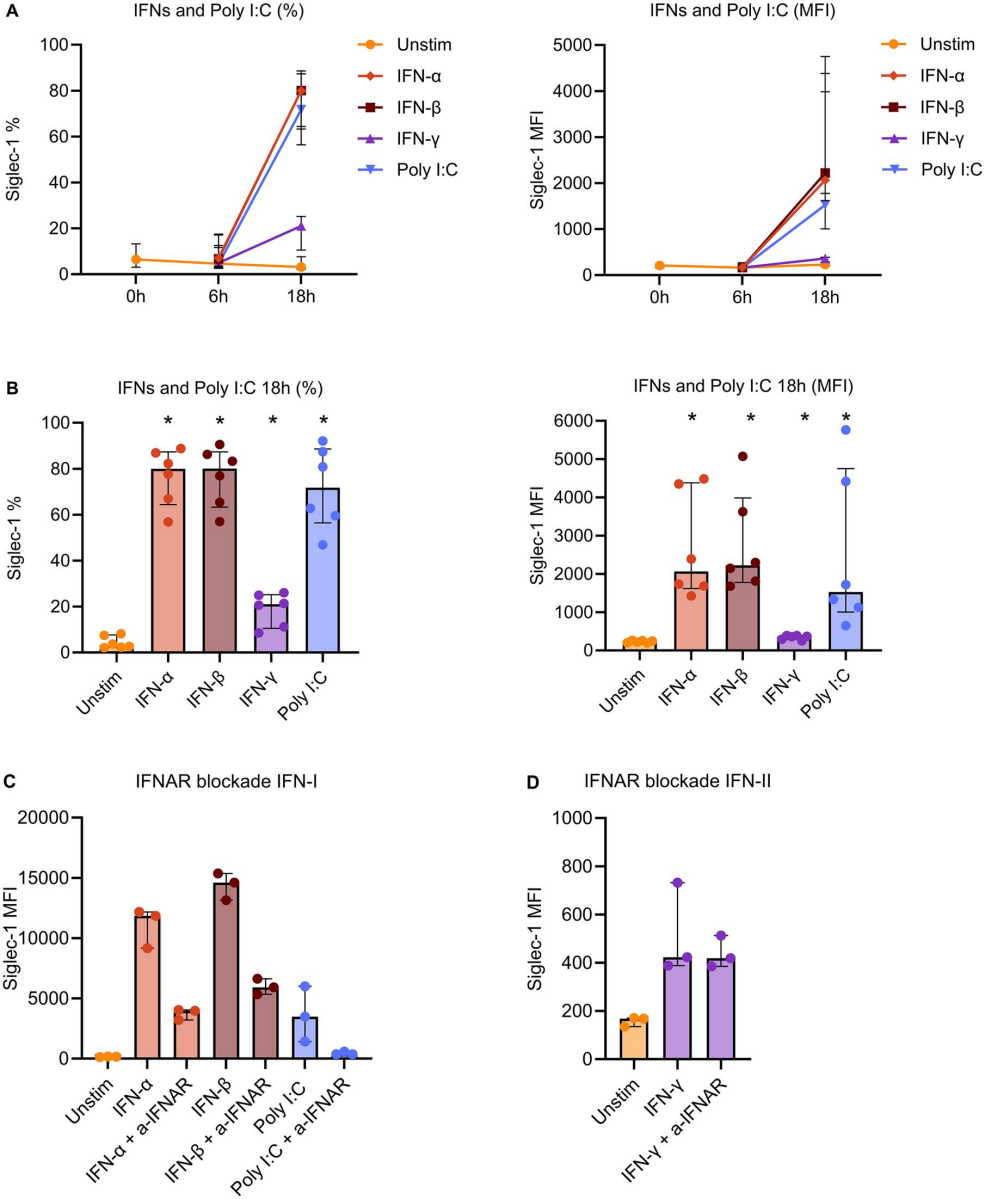
Siglec-1 is induced after 18 h by type I Interferons. (A) Healthy donor (HD) PBMCs (*N* = 6) were treated with IFN-α (1000 U/ml), IFN-β (1000 U/ml), IFN-γ (10 ng/ml) or TLR-3 agonist poly I:C (50 μg/ml) for 0 (ex vivo), 6 or 18 h (h). (B) Siglec-1 levels after 18 h are shown. (C + D) HD PBMCs (*N* = 3) were pre-incubated with an anti-IFNα/βR2 (a-IFNAR) blocking antibody (2 μg/ml) before stimulation with IFN-α (1000 U/ml), IFN-β (1000 U/ml), TLR-3 agonist poly I:C (50 μg/ml) (C) or IFN-γ (10 ng/ml) for 18 h (D). Siglec-1 expression was analyzed on CD14^+^ monocytes using flow cytometry and presented as both the percentage of Siglec-1^+^ cells within the viable CD14^+^ monocyte population (A + B, left panels) and median fluorescent intensity (MFI) of Siglec-1 on viable CD14^+^ monocytes (A + B, right panels and C + D). Medians with interquartile ranges are shown in all panels. Statistical significance was only tested for comparisons with *N* ≥ 5 per condition. *P < 0.05, comparison with unstimulated condition. TLR = toll-like receptor

To investigate the kinetics and stimuli driving Siglec-1 upregulation on monocytes, we cultured PBMCs from HDs with different types of IFNs (IFN-α, -β, -γ), TLR-agonists (Poly I:C, CpG-A ODN Class A, Imiquimod, LPS) and other cytokines (IL-1β, TNF-α) for different durations. TLR-agonists were included to mimic innate immune activation. Siglec-1 expression was low on HD monocytes *ex vivo* (0 h) but was significantly induced *in vitro* after 18 h stimulation with IFN-α, IFN-β and TLR-3 agonist Poly I:C, and to a much lesser extent by IFN-γ (Fig. 2A and B). Additionally, stimulation with TLR-7 and -9 agonists (Imiquimod and CpG-A ODN), also resulted in Siglec-1 expression after 18 h (Supplementary Fig. S2A and B, available at *Rheumatology* online). A minimal increase in the percentage of Siglec-1^+^ cells but not in MFI was observed in PBMCs stimulated with TNF-α. Conversely, LPS elicited a minimal increase in MFI without affecting the percentage of Siglec-1^+^ cells (Supplementary Fig. S2A and B, available at *Rheumatology* online). Stimulation with IL-1β did not induce Siglec-1 expression.

We also investigated Siglec-1 induction after 48 and 72 h cultures; however, we observed considerable variation between donors, probably due to an increased cell death of >50% (data not shown). We therefore continued with 18 h as cell culture duration for subsequent experiments.

To investigate whether induction of Siglec-1 by IFNs and Poly I:C was mediated through the IFN-α/β receptor (IFNAR), we incubated HD PBMCs with a monoclonal antibody blocking IFNAR2 1 h prior to stimulation. Siglec-1 induction by IFN-α, IFN-β and Poly I:C could be prevented by this blockade, whereas the (low) induction by IFN-γ could not (Fig. 2C and D). These findings confirm that type I IFNs induce Siglec-1 expression through binding of IFNAR and that TLR3 stimulation by Poly I:C leads to the production of type I IFN, which also induces Siglec-1 expression via IFNAR.

In conclusion, these findings confirm Siglec-1 as a marker that is rapidly upregulated on monocytes upon stimulation with type I IFNs.

### Siglec-1 induction can be inhibited in a dose-dependent manner by different JAKi

**Figure 3.**
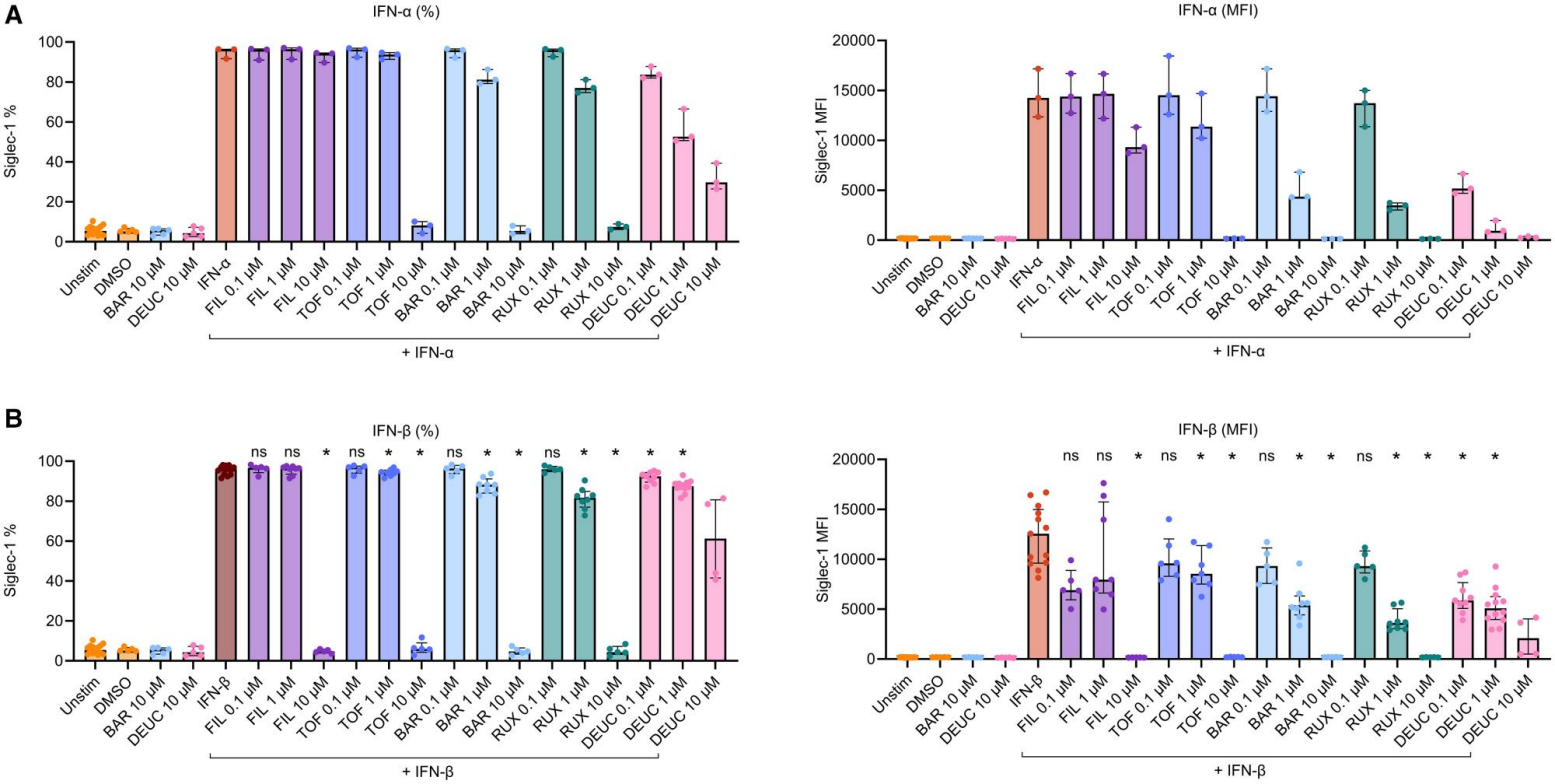
Dose-dependent inhibition by JAK inhibitors of IFN-α- and IFN-β-mediated Siglec-1 induction. Healthy donor (HD) PBMCs were pre-incubated for 1 h with 0.1–10 μM filgotinib (FIL), 0.1–10 μM tofacitinib (TOF), 0.1–10 μM baricitinib (BAR), 0.1–10 μM ruxolitinib (RUX) or 0.1–10 μM deucravacitinib (DEUC) before stimulation with IFN-α (1000 U/ml) (*N* = 3) (A) or IFN-β (1000 U/ml) (*N* = 4–13) (B) for 18 h. Control conditions were included (unstimulated, DMSO-only, BAR-only, DEUC-only, IFN-only). Siglec-1 expression was analyzed on CD14^+^ monocytes using flow cytometry and presented as both the percentage of Siglec-1^+^ cells within the viable CD14^+^ monocyte population (left panels) and median fluorescent intensity (MFI) of Siglec-1 on viable CD14^+^ monocytes (right panels). Medians with interquartile ranges are shown. Statistical significance was only tested for comparisons with *N* ≥ 5 per condition. *P < 0.05, comparison with IFN-β-only condition. DMSO = dimethylsulphoxide

To examine whether induction of Siglec-1 can be inhibited by JAKi, HD PBMCs were incubated with different concentrations of filgotinib, tofacitinib, baricitinib, ruxolitinib and deucravacitinib 1 h before adding IFN-α or -β. We found a dose-dependent inhibition of IFN-α- and IFN-β-mediated Siglec-1 induction for all JAKi (Fig. 3). The reduction in Siglec-1 MFI was more pronounced than the decrease in Siglec-1 percentage, indicating that the expression of Siglec-1 on cell membranes was decreased but was not normalized on all cells (illustrative FACS plot in Supplementary Fig. S1, available at *Rheumatology* online). Inhibition of IFN-α- *vs* IFN-β-mediated Siglec-1 induction by JAKi was similar, except for filgotinib at a concentration 10 µM, which could only moderately inhibit Siglec-1 induction (MFI) by IFN-α, but completely inhibited Siglec-1 induction (both MFI and %) by IFN-β. In contrast, deucravacitinib appeared more potent in inhibiting IFN-α-mediated Siglec-1 induction compared with IFN-β. Complete inhibition was also observed in conditions with 10 µM of other JAKi. While a slightly higher cell death was observed in some of these 10 µM conditions, the median cell viability did not go below 62% (control condition with deucravacitinib 10 µM) (Supplementary Fig. S3, available at *Rheumatology* online). Taken together, our data show that Siglec-1 induction by IFN-α or -β can be inhibited by JAKi, with outcomes varying depending on the type of stimulation and the type and concentration of the JAKi.

### Plasma or serum from patients with (J)DM, JSLE and COVID-19 induce Siglec-1 on healthy monocytes and this can be inhibited by IFNAR blockade and JAKi

To evaluate the suitability of the assay for investigating patient-specific responses, we conducted co-culture experiments by combining PBMCs from HDs with plasma or serum derived from patients diagnosed with diverse type I IFN-related diseases, rather than directly supplementing the culture with exogenous IFN-α or IFN-β. We used plasma samples obtained at diagnosis (*N* = 8) and during clinical remission (*N* = 2, paired) from JDM patients, serum samples obtained at diagnosis from DM patients (*N* = 6), plasma samples obtained at diagnosis from JSLE patients (*N* = 2) and plasma samples from adult patients with severe COVID-19 infection, obtained on the day of hospitalization (*N* = 2). Plasma samples from HDs (distinct from those from whom PBMCs were obtained) were collected to serve as a control group. All patients were treatment-naïve at moment of sampling at diagnosis. Further clinical characteristics of patients and HDs are depicted in Table 1.

**Figure 4.**
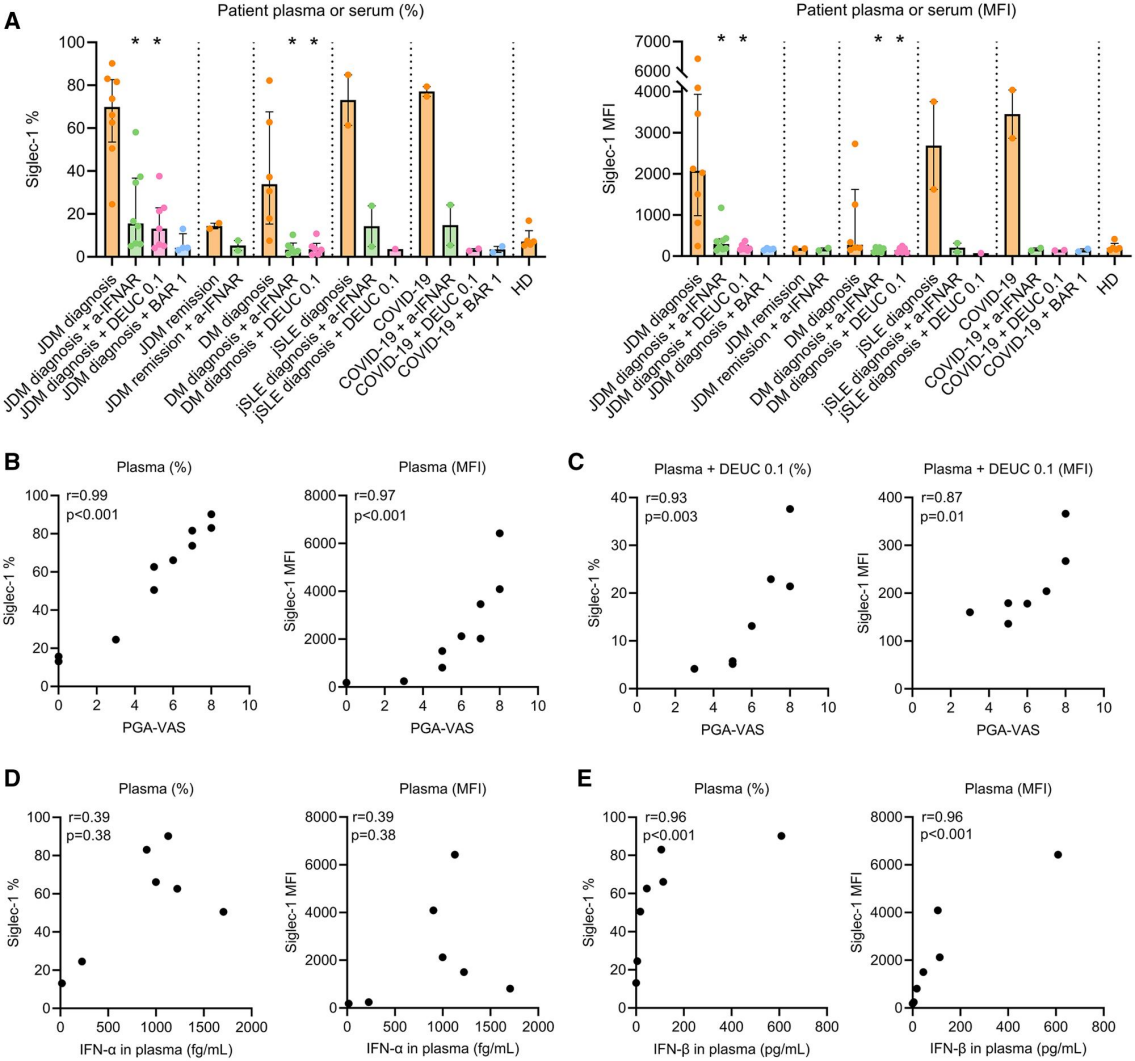
Siglec-1 is induced by plasma or serum from patients with IFN-driven diseases and correlates with clinical disease activity and IFN-β in plasma. Healthy donor (HD) PBMCs (*N* = 3) were treated for 18 h with plasma or serum from patients or from (other) HDs (17% v/v), with or without 1 h pre-incubation with 2 μg/ml anti-IFNα/βR2 (a-IFNAR) blocking antibody, 0.1 μM deucravacitinib (DEUC) or 1 μM baricitinib (BAR). Siglec-1 expression was analyzed on CD14^+^ monocytes using flow cytometry and presented as both the percentage of Siglec-1^+^ cells within the viable CD14^+^ monocyte population (left panels) and median fluorescent intensity (MFI) of Siglec-1 on viable CD14^+^ monocytes (right panels). Pan-IFN-α and IFN-β levels were analyzed in JDM plasma samples by a multiplexed Quanterix homebrew Simoa assay (digital ELISA). (A) Siglec-1 expression presented in medians with interquartile ranges. Statistical significance was only tested for comparisons with *N* ≥ 5 per condition. *P < 0.05, comparison with plasma/serum-only condition. (B and C) Spearman correlations between PGA-VAS score and Siglec-1 expression induced by JDM plasma without (B) and with (C) pre-incubation with 0.1 μM deucravacitinib (DEUC). (D and E) Spearman correlations between JDM plasma-induced Siglec-1 expression and IFN-α (D) and IFN-β (E) plasma levels. PGA-VAS = Physician’s Global Assessment of overall disease activity measured on a Visual Analogue Scale (0–10)

Co-culturing of HD PBMCs with plasma from JDM patients obtained at diagnosis resulted in a potent and significant induction of Siglec-1. This was observed both in the percentage of Siglec-1^+^ monocytes (median 69.9%, IQR 53.5–82.7 with JDM plasma *vs* median 7.1%, IQR 5.5–12.2 with HD plasma; *P* = 0.002) and MFI (median 2073, IQR 983–3934 with JDM plasma *vs* median 192, IQR 79–309 with HD plasma; *P* = 0.003) (Fig. 4A). Pre-treatment with either the anti-IFNAR antibody, deucravacitinib (0.1 µM) or baricitinib (0.1 µM) markedly reduced Siglec-1 levels, which were close or similar to those observed in the condition with HD plasma. Plasma obtained during clinical remission from two JDM patients (both still receiving methotrexate) resulted in a marginal increase in the percentage of Siglec-1^+^ monocytes, which could be reduced through anti-IFNAR blockade. Co-culturing with plasma from JSLE patients and COVID-19 patients also induced Siglec-1 expression, which could similarly be inhibited. Additionally, serum from treatment-naïve DM patients also induced Siglec-1 expression (% > MFI), although to a lesser extent than the other disease groups. To investigate whether this difference was due to the use of serum instead of plasma, we compared paired samples from JSLE patients. The induction of Siglec-1 by plasma and serum was highly similar (Supplementary Fig. S4, available at *Rheumatology* online). However, we noted that fewer cells were available for analysis in the conditions with plasma compared with serum, possibly due to differences in cell viability and/or cell adherence to the plate.

Given the observed variability in Siglec-1 induction by JDM plasma obtained at diagnosis, we investigated its correlation with the level of global disease activity at the moment of sampling, as assessed by the treating physician (PGA-VAS score). We found an almost perfect correlation between the level of global disease activity and the level of Siglec-1 induced by JDM plasma (Siglec-1%: rs = 0.99, *P* < 0.001; Siglec-1 MFI: rs = 0.97, *P* < 0.001; Fig. 4B). Additionally, we observed a strong correlation between the level of global disease activity and the level of (remaining) Siglec-1 when pre-treated with deucravacitinib (0.1 µM), suggesting an association between higher disease activity and suboptimal inhibition (Siglec-1%: rs = 0.93, *P* < 0.003; Siglec-1 MFI: rs = 0.87, *P* = 0.01; Fig. 4C). When evaluating Siglec-1 expression induced by JDM plasma or DM serum in relation to myositis-specific autoantibody profiles, we observed the highest levels in anti-NXP2^+^ (*n* = 5), anti-MDA5^+^ (*n* = 1) and anti-TIF1y^+^ (*n* = 4) patients (Supplementary Fig. S5, available at *Rheumatology* online). However, the limited sample size prevents robust conclusions.

Furthermore, we measured pan-IFN-α and IFN-β levels in a subset of the JDM plasma samples, and found a strong correlation between plasma-induced Siglec-1 expression and IFN-β plasma levels (Siglec-1% and MFI: rs = 0.97, *P* < 0.001) (Fig. 4D and E). Removal of one outlier with very high IFN-β levels did not substantially alter the correlation (Siglec-1% and MFI: rs = 0.94, *P* = 0.005, Supplementary Fig. S6, available at *Rheumatology* online). In contrast, no significant correlation was observed with pan-IFN-α plasma levels (Siglec-1% and MFI: rs = 0.4, *P* = 0.4). These results strongly suggest that Siglec-1 is induced by IFN-β rather than IFN-α in plasma from JDM patients. As expected, IFN-β levels correlated with global disease activity, while pan-IFN-α levels did not (Supplementary Fig. S7, available at *Rheumatology* online).

Taken together, plasma or serum from treatment-naïve JDM patients and those with other type I IFN-related diseases induce Siglec-1 expression on healthy monocytes. This induction can be effectively inhibited by anti-IFNAR blockade and JAKi. Additionally, Siglec-1 levels induced by JDM patient plasma show a strong correlation with global disease activity and IFN-β plasma levels. These findings highlight the assay’s potential for studying patient-specific type I IFN-mediated responses and point to IFN-β as the main driver of Siglec-1 expression in JDM.

## Discussion

Although a growing number of studies have investigated Siglec-1 as an *ex vivo* biomarker in IFN-driven autoimmune diseases, our current study demonstrates its utility as an accurate *in vitro* read-out for type I IFN-mediated responses [11, 14–18, 27]. We found Siglec-1 to be rapidly upregulated on monocytes, easy to measure, and specific for type I IFN stimulation. Our findings are consistent with those published by York *et al.* who showed induction of Siglec-1 on healthy control CD14^+^ PBMCs exposed to IFN-α, TLR-3, TLR-7 or TLR-9 agonists, an effect that could be inhibited by B18R, a soluble type I IFN receptor [18]. Several *in vitro* studies have reported IFN-α-mediated upregulation of Siglec-1, primarily in an infectious disease context [28, 29]. In this study, we demonstrate that IFN-β, too, induces Siglec-1, aligning with findings from Dupont *et al.* in a tuberculosis model [30].

Notably, we found a strong correlation between the level of Siglec-1 induction on healthy monocytes co-cultured with JDM patient plasma and IFN-β plasma levels. In contrast, no correlation was observed with IFN-α, which was present at lower levels than IFN-β in plasma. This suggests that the type I IFN signature in JDM patients is predominantly driven by IFN-β. While this could simply reflect its higher abundance, previously shown by others and potentially linked to RIG-I stimulation, emerging evidence suggests that IFN-β may exert more pathogenic effects than IFN-α, particularly in muscle tissue [31–35]. In vitro, IFN-β, rather than IFN-α, has been shown to impair myotube formation and reduce contractile force and twitch kinetics in myobundles [34, 35]. Clinically, strong correlations have been observed between IFN-β plasma levels and clinical disease scores, while IFN-α levels only correlated with disease scores in anti-MDA5+ patients, a subtype of JDM with distinct clinical and pathological features [36]. While the underlying mechanisms remain unclear, the differences may be attributed to the higher binding affinity and greater stability of IFN-β with IFNAR1 and IFNAR2 compared with IFN-α, leading to more sustained and potent signalling [35, 37, 38].

Regarding the inhibition of these type I IFN subtypes, we observed that the inhibition of IFN-α- and IFN-β-mediated Siglec-1 induction by various JAKi followed a similar pattern. However, IFN-β-mediated induction was more effectively inhibited by filgotinib, a JAK1 inhibitor and less effectively by deucravacitinib, a TYK2 inhibitor, when compared with IFN-α-mediated induction. These findings suggest that IFN-β signalling may be more dependent on JAK1 compared with IFN-α, a notion supported by the observation that TYK2-deficient cells retain partial responsiveness to IFN-β, but not IFN-α [38]. Understanding these differences may have important therapeutic implications for selectively modulating type I IFN responses in diseases such as JDM.

Independent of the type of IFN stimulation, we observed variability in inhibitory efficacy based on the type of JAKi used and its concentration. For example, deucravacitinib was more potent than filgotinib or tofacitinib (a JAK1/3 and partial JAK2 inhibitor) in inhibiting IFN-α-mediated Siglec-1 induction at a concentration of 1 μM. This may be attributed to deucravacitinib being a highly selective allosteric TYK2 inhibitor targeting the regulatory pseudokinase domain of TYK2, whereas the classical JAK1-3 inhibitors act by competing at the ATP-binding catalytic site, which is highly conserved across many kinases [39]. These ATP-competitive inhibitors likely become less selective at higher doses, which may also be reflected in our *in vitro* data, where the JAK1-3 inhibitors completely blocked Siglec-1 induction at high concentrations of 10 μM. Filgotinib was unable to achieve complete inhibition under IFN-α conditions, which may correspond to the relatively high doses (200 mg/day) typically required in clinical practice. Though off-target binding raises safety concerns, the clinical impact of selectivity differences among JAKi remains uncertain. Translating *in vitro* selectivity to *in vivo* settings is challenging due to variability in cell lines, read-outs and dosages, as well as pharmacodynamic and pharmacokinetic factors like drug metabolism and tissue sensitivity. Head-to-head trials are ultimately needed but difficult to conduct, especially in rare diseases and paediatric conditions. In JDM, the use of JAKi has primarily been reported in case studies and series with tofacitinib, ruxolitinib and baricitinib—the only JAKi approved for paediatric use in Europe [19]. An assay using patient-derived samples to guide JAKi selection would be valuable, along with a reliable and easy-to-use read-out for clinical trials.

The induction of Siglec-1 on healthy monocytes following incubation with patient plasma, along with its correlation with clinical disease activity, highlights the potential of our assay for assessing patient-specific IFN responses. Given that these responses could be inhibited by various IFN pathway inhibitors, the next logical step is to evaluate whether the assay can aid in stratifying patients for JAKi treatment and selecting the most effective JAK inhibitor. Furthermore, the induction of Siglec-1 by plasma from patients with DM, JSLE and COVID-19 indicates that the assay may have broader applicability across various IFN-mediated diseases.

Limitations of this study include its relatively small sample size. Larger studies are needed to confirm our observations and explore inter-individual variability. Additionally, while our *in vitro* model primarily focuses on type I IFN activation—a central pathway in JDM—it does not fully capture the complexity of *in vivo* immune responses, which involve pharmacokinetic and pharmacodynamic factors, tissue-specific effects and broader immune interactions, including vasculopathy and adaptive immunity. Although these factors may modulate Siglec-1 expression, our findings—including the minimal to no Siglec-1 induction by TNF-α, LPS and IL-1β—highlight IFN-induced Siglec-1 expression as a prominent pathway, and Siglec-1 as a reliable readout of type I IFN activity. While translating *in vitro* findings to *in vivo* settings is inherently challenging, our chosen JAKi concentration range (0.1, 1.0 and 10 µM) includes clinically observed peak plasma levels (0.05–5 µM, depending on JAKi), ensuring relevance while also allowing assessment of dose-dependent effects and maximal inhibition [40–44]. Finally, the majority of the HDs in this study were not age-matched. Although cytokine levels and Siglec-1 expression may differ between healthy adults and children, our previously published data on JDM-associated cytokine profiles and Siglec-1 expression in both age groups suggest that age is unlikely to have significantly influenced our results [11, 45].

In conclusion, Siglec-1 is a reliable *in vitro* marker for type I IFN responses, and its induction can be effectively inhibited by JAKi, depending on the type of stimulation, JAKi and concentration. Our findings suggest that IFN-β plays a predominant role in driving Siglec-1 induction in JDM *in vivo*. This assay holds promise for studying patient-specific IFN responses and could provide a biological foundation for precision treatment in IFN-driven diseases.

## Supplementary Material

keaf227_Supplementary_Data

## Data Availability

All data and protocols of this study are available to the scientific community upon reasonable request.
