# Supplementary material for: Targeting interferon responses in juvenile dermatomyositis: Siglec-1 as an in vitro biomarker for JAK inhibitor efficacy
Source: Rheumatology (Oxford). 2025 May 15;64(9):5132–41. doi: 10.1093/rheumatology/keaf227 (PMC12407234; doi:10.1093/rheumatology/keaf227)
Supplement: keaf227_Supplementary_Data [file keaf227_supplementary_data.docx]

**
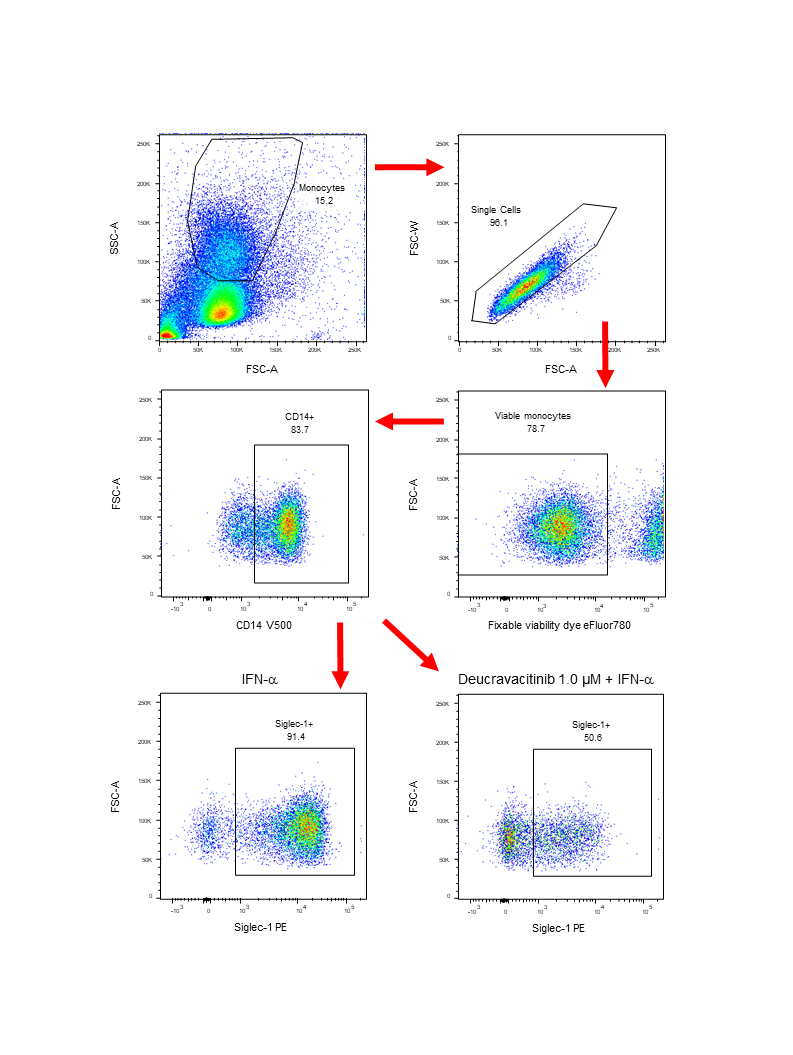
**

**Supplementary Figure S1. Gating strategy and illustrative FACS plots.** Siglec-1 expression on CD14^+^ PBMCs acquired from a healthy donor and stimulated with IFN-α (1000 U/mL) (lower left plot) or pre-incubated with 1.0 μM deucravacitinib and stimulated with IFN-α (1000 U/mL) (lower right plot) *in vitro*. Gating strategy: monocyte population identified in FSC/SSC plot, followed by gating on single cells, viable cells, CD14⁺ cells, and Siglec-1⁺ cells. The Siglec-1 gate was set based on fluorescence-minus-one (FMO) controls.


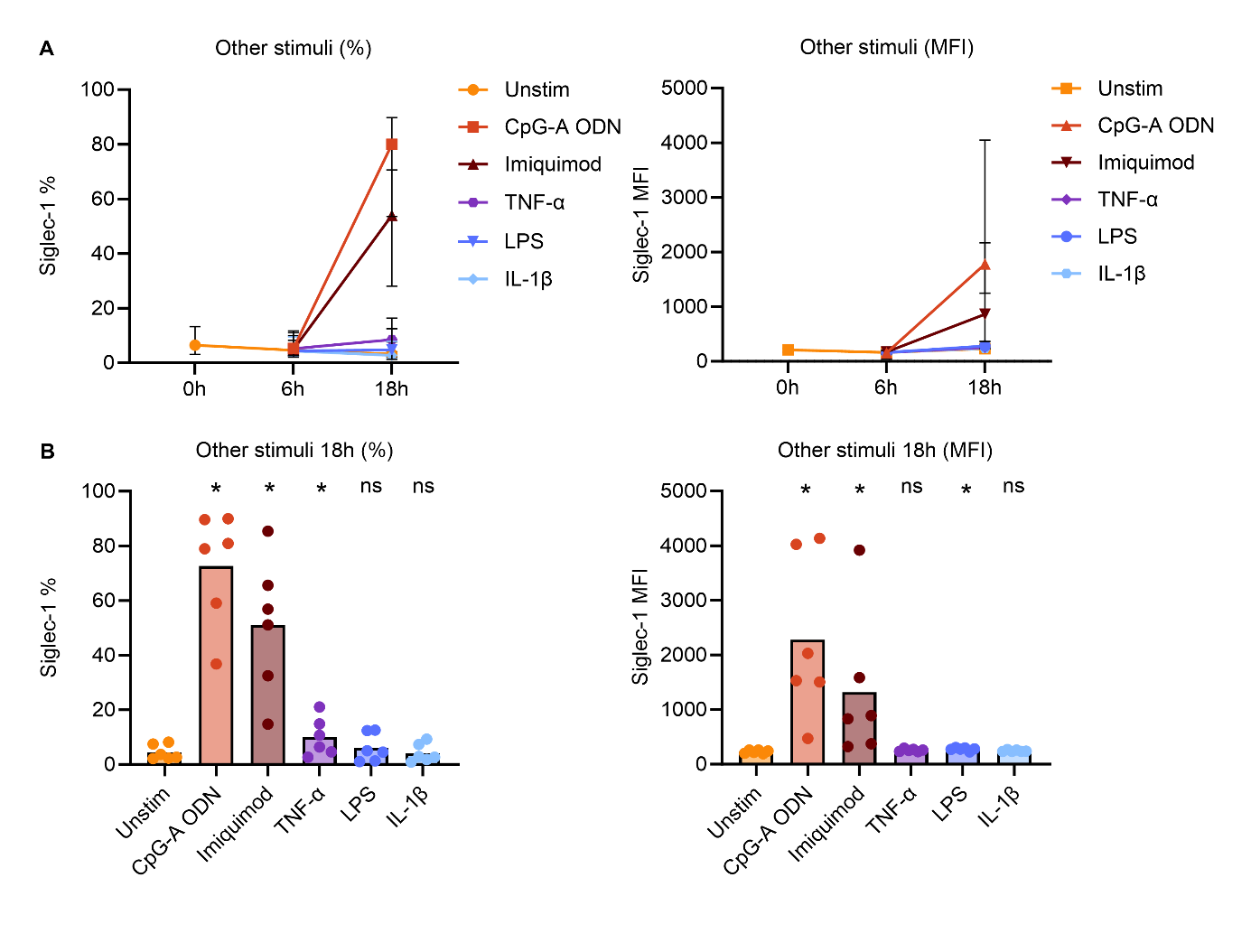

**Supplementary Figure S2. Siglec-1 induction over time by other stimuli. (A)** Healthy donor (HD) PBMCs (N=6) were treated with TLR-9 agonist CpG-A ODN (1 μg/ml), TLR-7 agonist imiquimod (R837) (5 μg/ml), TNFα (10 ng/ml), TLR-4 agonist LPS (10 ng/ml), or IL-1β (10 ng/ml) for 0 (ex vivo), 6, or 18 hours (h). **(B)** Median Siglec-1 levels after 18 h are shown. Siglec-1 expression was analyzed on CD14^+^ monocytes using flow cytometry and presented as both the percentage of Siglec-1^+^ cells within the viable CD14^+^ monocyte population (left panels) and median fluorescent intensity (MFI) of Siglec-1 on viable CD14^+^ monocytes (right panels). Medians with interquartile ranges are shown. ns = not significant, *p < 0.05, comparison with unstimulated condition. IFN = interferon, TLR = toll-like receptor.

**
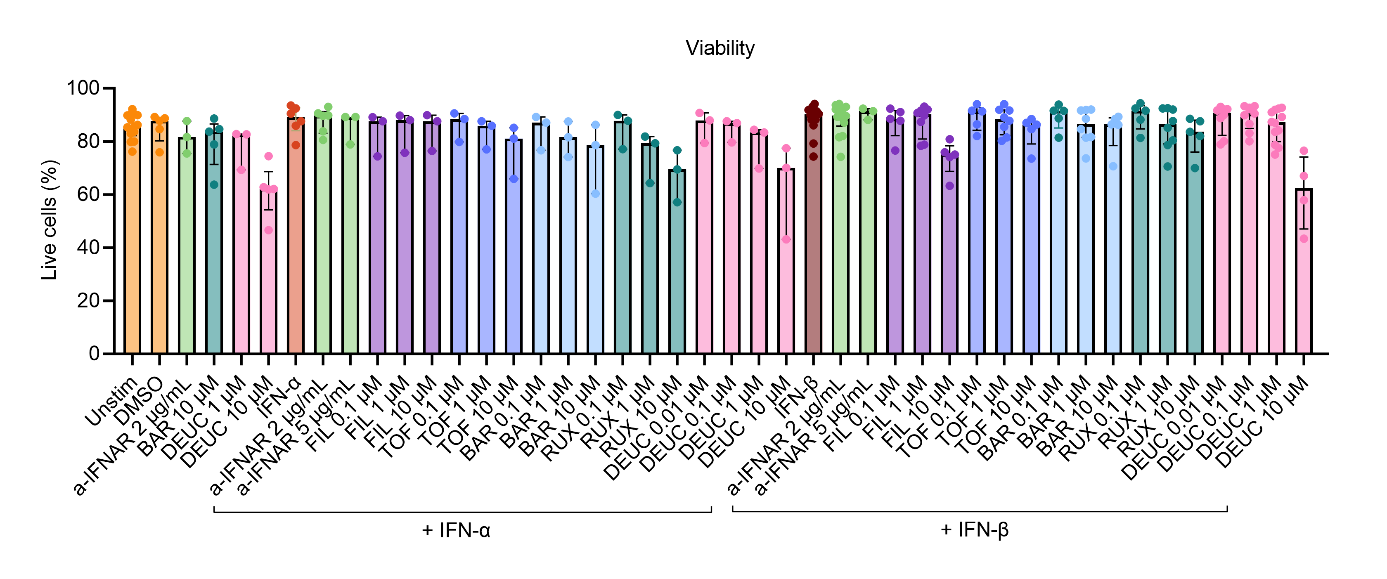

Supplementary Figure S3. Cell viability.** Healthy donor (HD) PBMCs were subjected to different treatment conditions. Cell viability was assessed using fixable viability dye eFluor780 (eBioscience™) and flow cytometry and is presented as the percentage of viable cells within the monocyte gate, as defined in the forward/side scatter plot. Medians with interquartile ranges are shown. DMSO = dimethylsulfoxide, IFNAR = IFNα/βR2, BAR = baricitinib, DEUC = deucravacitinib, IFN = interferon, FIL = filgotinib, TOF = tofacitinib, RUX = ruxolitinib.

**
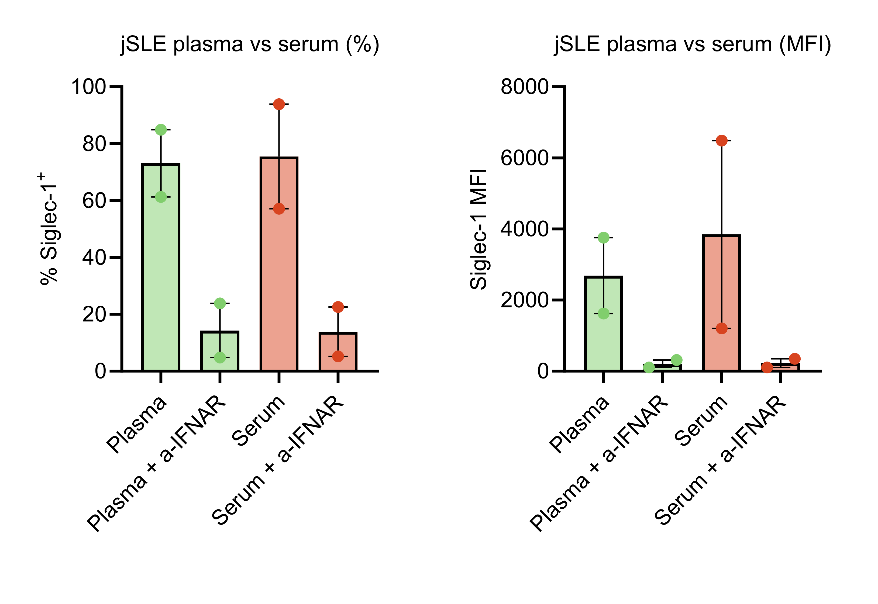

Supplementary Figure S4. Siglec-1 induction by plasma versus serum from jSLE patients.** Healthy donor (HD) PBMCs (N=2) were treated for 18 hours with plasma or serum from patients with juvenile systemic lupus erythematosus (jSLE) (17% v/v), with or without 1 hour pre-incubation with 2 μg/ml anti-IFNα/βR2 (a-IFNAR) blocking antibody. Siglec-1 expression was analyzed on CD14^+^ monocytes using flow cytometry and presented as both the percentage of Siglec-1^+^ cells within the viable CD14^+^ monocyte population (left panel) and median fluorescent intensity (MFI) of Siglec-1 on viable CD14^+^ monocytes (right panel). Medians with interquartile ranges are shown.


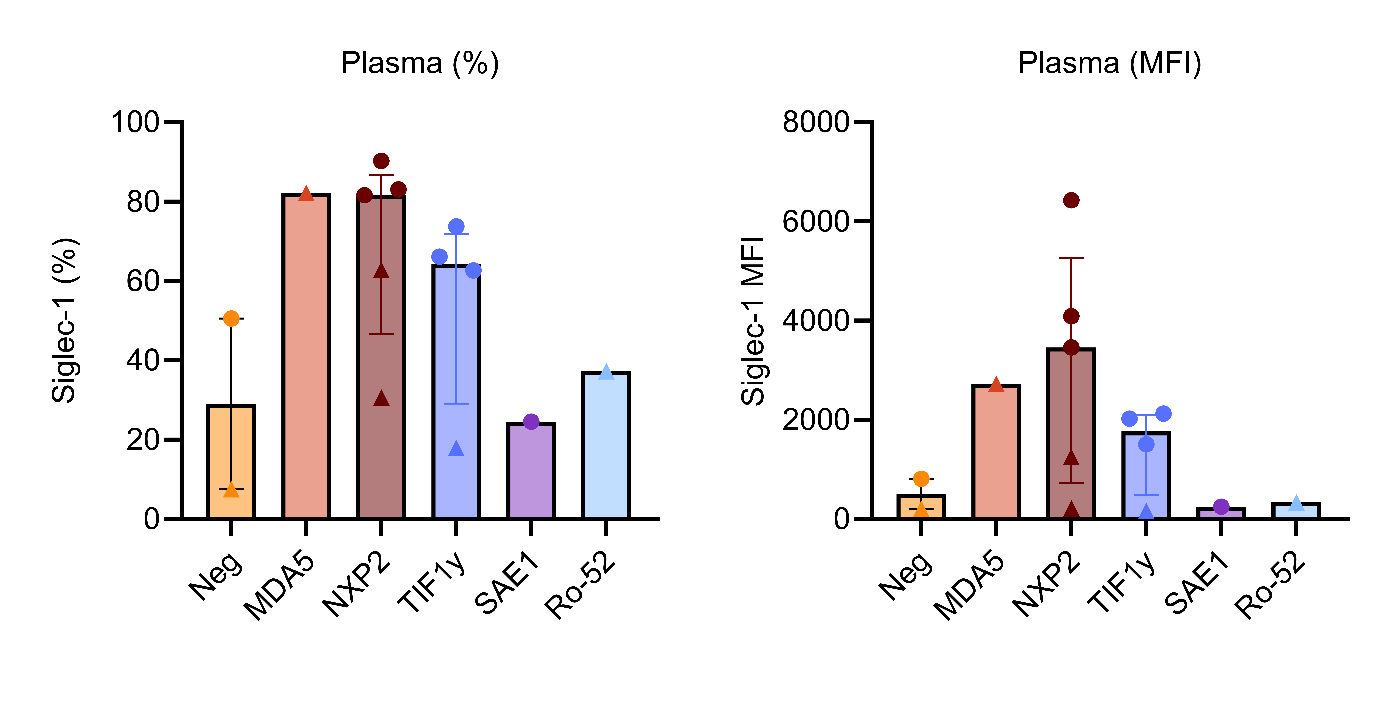

**Supplementary Figure S5. Siglec-1 expression induced by plasma or serum from (J)DM patients across MSA subtypes.** Siglec-1 expression is shown as the percentage of Siglec-1^+^ cells within the viable CD14^+^ monocyte population (left panel) and as the median fluorescence intensity (MFI) of Siglec-1 on viable CD14^+^ monocytes (right panel). Data represent plasma-induced Siglec-1 expression in JDM patients (filled circles) and serum-induced expression in DM patients (filled triangles). Only data from plasma samples obtained at JDM diagnosis are shown. MSA = myositis-specific autoantibody.


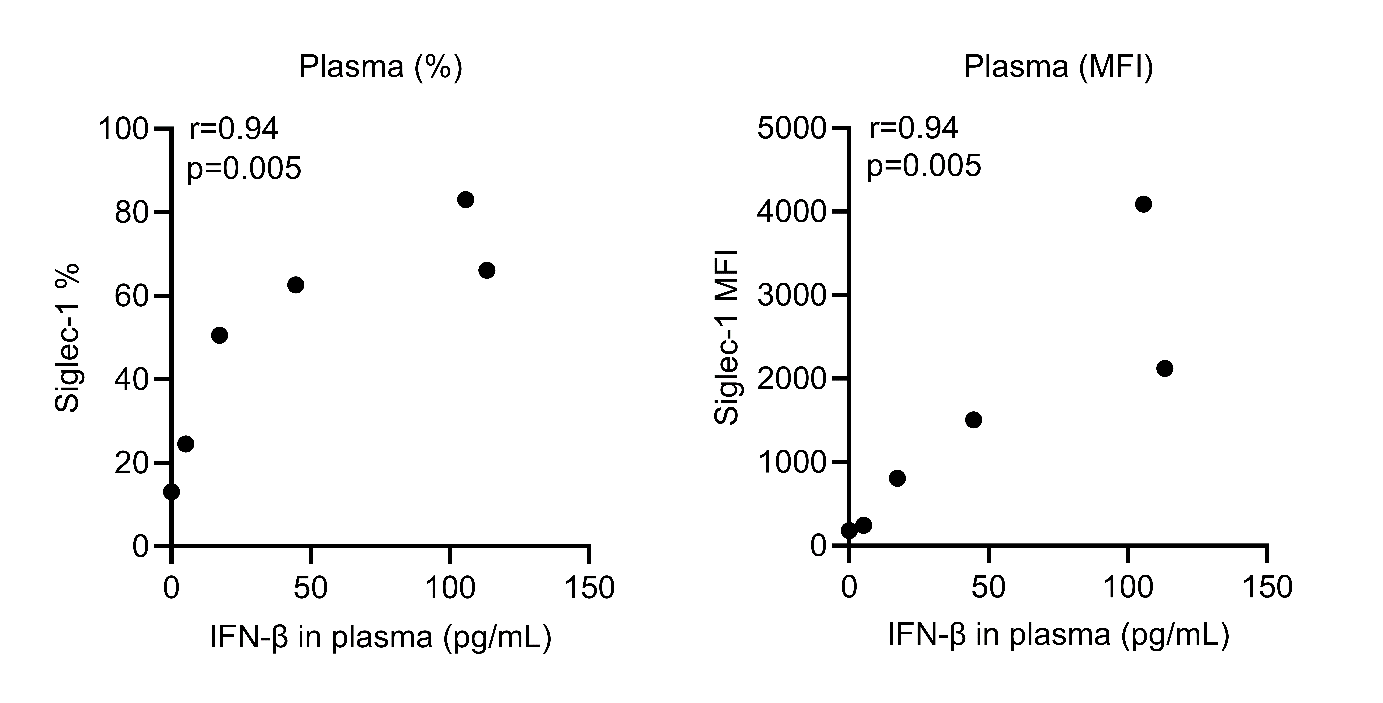


**Supplementary Figure S6. Spearman correlation between JDM plasma-induced Siglec-1 expression and IFN-β plasma levels, without outlier.** The graphs contain the same data as Figure 4E, but with the removal of one outlier with very high IFN-β levels.


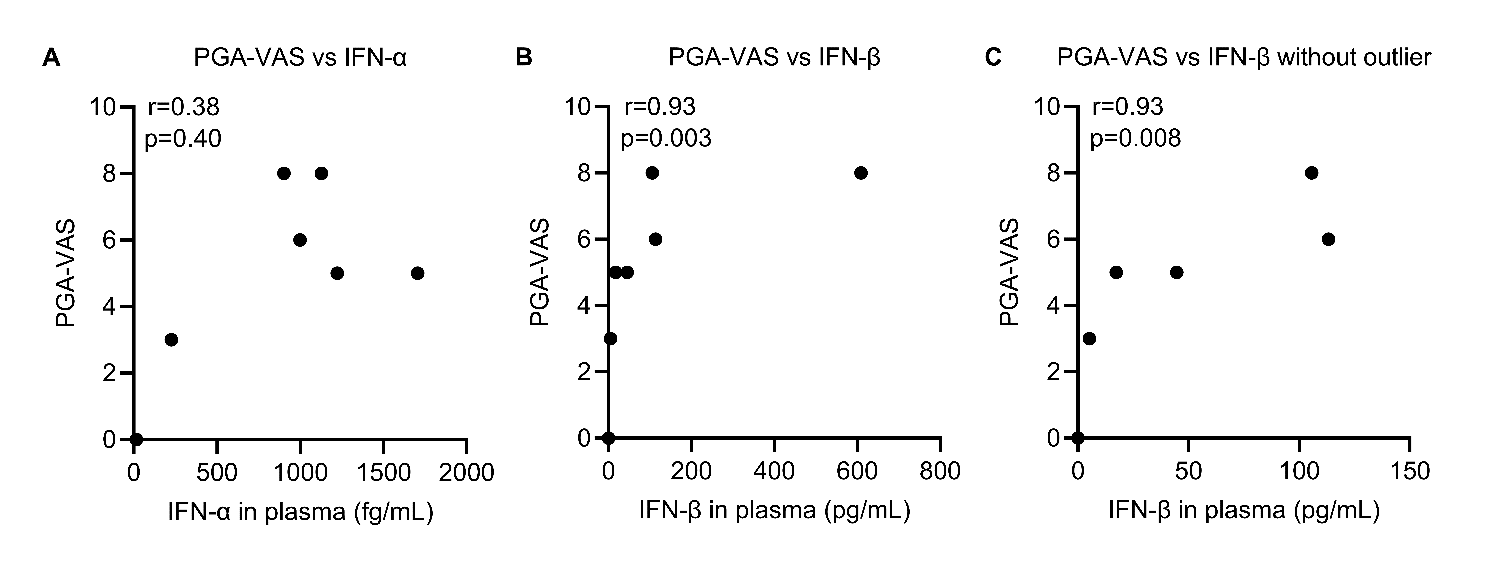

**Supplementary Figure S7. Correlations between PGA-VAS score and IFN plasma levels.** Spearman correlations between PGA-VAS score and IFN-α **(A)**, IFN-β **(B)**, and IFN-β plasma levels without outlier **(C)**. Pan-IFN-α and IFN-β levels were analyzed in JDM plasma samples by a multiplexed Quanterix homebrew Simoa assay (digital ELISA). PGA-VAS = Physician’s Global Assessment of overall disease activity measured on a Visual Analogue Scale (0-10).
